# Eighty Cases of Lupus Vulgaris
1Read before the Bristol Medico-Chirurgical Society, December 12th, 1906.


**Published:** 1907-06

**Authors:** W. Kenneth Wills

**Affiliations:** Physician in Charge of the Skin Department, Bristol General Hospital.


### EIGHTY CASES OF LUPUS VULGARIS.[1]

BY

W. KENNETH WILLS, M.D.,

*Physician in Charge of the Skin Department, Bristol General Hospital.*

---

ONE of the most difficult problems in medicine is the correct estimation of the value of any new method of treatment which is not absolutely specific. Not only is enthusiasm liable to run riot at the incidence of a series of successful cases, but the reverse may also occur, and undue depreciation of the value of a useful method be engendered by an initial series of *un*successful cases.

[1] Read before the Bristol Medico-Chirurgical Society, December 12th, 1906.

In attempting to make a personal estimate of the value of radio-therapeutic methods in the treatment of lupus vulgaris, several difficulties presented themselves at the offset.

It was claimed for the Finsen Light treatment that the rays which were curative in property were those rays of short but rapid wave-length in the blue and violet, and beyond the violet end of the spectrum. But it was soon shown that the penetration of these rays was of the smallest character, while I myself have shown that rays of the less therapeutic value in the spectrum pass with ease through the body. The first difficulty I met with was in choosing the cases which would be best suited for the treatment, out of the numerous cases which presented themselves at the department. For the most part these were of the most unpromising nature for any new method of treatment—the subjects of old-standing disease, in some cases over forty years in duration, with a history of numerous operations, many of whom presented deep and extensively scarred lupus of the integument with free involvement of mucous membranes.

Secondly, it soon became obvious that there were nearly as many types of the disease as there were patients to suffer from them. And success or failure in one case c6uld hardly be claimed as experience in the next.

Thirdly, the patients were largely of the " night-bird " order, creeping out after night-fall or swathing up their disfigurements in light-proof veils and wraps, leading thus an unhealthy and unnatural existence, often in great want owing to the inability to obtain even the scantiest livelihood.

As an instance of how this factor has a bearing on the estimation of value of the treatment, I may cite the case of a man who was obtaining the scantiest livelihood by selling newspapers—his face always hidden under a scarf—poor to destitution, but independent. I attempted his relief by means of radio-therapy with no good result. Some years after I happened to meet him, and urged him again to come for treatment. He came, and in order to do so, went into the workhouse, and obtained there better diet, regime and attention. Instantly we obtained a most promising effect, and the amelioration is continuing in a most remarkable way.

Although aware of these difficulties, I thought that I would at first restrict the treatment of the exterior at any rate to the radio-therapeutic methods alone, and attempt to estimate the value *per se* of the radio-therapeutics as a specific. If some cases seem to have been a very long while under treatment, it must be remembered that until quite recently no other treatment was used in conjunction with the light, that many of these were old scarred cases, and some of them cases on which the Finsen treatment was merely wasted.

The first case of lupus was treated in the department in December, 1901. Up till the end of 1905 we have a record of over eighty cases of lupus. Besides these there have been not a few instances of other manifestations of cutaneous tubercular disease, such as scrofulodermia, tubercular abscesses, verruca necrogenica and the allied verrucous condition on the hands of butchers, which is in all probability tubercular. These might very well form a separate note, since they have been extremely ready to respond to radio-therapy, more so than lupus vulgaris, but I have omitted them from this collection.

The variations in type of lupus are numerous but not always well defined, one type merging into another according to the reaction of the patient to the disease. I have divided off the following :—

1. Extensive erythematous forms, spreading widely and rapidly at intervals, sometimes more or less symmetrical in position, depending upon unknown factors for their delimitation. Perhaps nerve areas are roughly mapped out ; certainly the hairy areas seem to offer great resistance.

2. Nodular forms, with very slight erythema occupying one or more areas, with no suggestion of nerve area or symmetry ; no tendency to ulceration, and of very slow growth.

3. Hypertrophic forms (lupus exedens), with high-raised granulation tissue, of rapid development and spread.

4. Ulcerative forms, where ulceration is the chief feature of the case, not merely an incident in the case.

5. Verrucous forms, indolent in growth, very chronic and resistant.

6. Oedematous forms, with lymph-stasis out of all portion to the amount of disease, but the cutaneous exanthem showing definite " apple-jelly " nodules.

7. Sclerodermic forms.

As I have said, these types may overlap, it being difficult to classify some cases under any of these headings. But in general the case is true to its own type, whatever that type may be. If it is hypertrophic on the face, it will be hypertrophic on the hand ; if nodular on the thigh, it will be nodular on the ear ; and so on. It is rare to find also lupus in one patch while there is some indefinite scrofulodermia elsewhere. It would appear that the type of disease is the expression of the tissue resistance rather than the virulence of the strain. At the same time it is quite rare to find phthisis in a lupus patient. It is present in a very chronic fibroid form in three out of our eighty cases—3.75 per cent. Other forms of tubercle may be present, though not commonly. Tubercular dactylitis in three cases—3.25 per cent. Hip-disease (old) in one—1.25 per cent. Enlarged glands in several.

Lacrymal dacryocystitis and abscess is fairly frequent when the nasal mucous membrane is attacked. It is of interest to note how many patients with lupus of the face have attending infection of the nasal mucous membrane. Indeed it is not infrequent for the patient to admit having had a liability to epistaxis and coryza or blocked nostril for a long period before lupus has developed externally.

The various types behave differently to the radio-therapeutic measures. The extensive erythematous forms are very difficult to treat. It requires long-continued patient care to make any headway at all with the light, and with the X-rays many and frequent applications are necessary before the erythema dies away and scar tissue is revealed.

As soon as this paling of the area is observed, it will be noticed that broad bands of white supple scar tissue leave between them islands of erythematous material, which gives the diffuse staining characteristic of this kind of lupus through the diascope. The X-ray treatment must be continued for a long while after this, and it is this feature which opens up an avenue of danger in persons past the middle age. Long-continued X-ray treatment in a case where scarring is extensive, and is cutting off islands of inflammatory material, may be followed by the development of carcinoma. It is recognised that carcinoma may develop on an untreated lupus, the reparative processes of nature pursuing the same course, but there is obviously a danger of raising these undesirable statistics by the *too* rapid encouragement of this healing process. Epithelioma developed on the lip in one very extensive case of this form of the disease, and in spite of extensive removal, which Mr. Hey Groves was good enough to undertake, the patient went from bad to worse and died.

It is jumping to conclusions to state with any degree of definiteness that this was produced by the ray-treatment, but having watched the case for years there is a haunting conviction that the treatment was a contributary cause.

Perhaps one attempted to do too much. In the initial stages of the treatment the light produced a great amelioration of his distressing condition. Later, as the light did not seem to be curing any part, I added the X-rays. Improvement and the scarring of large areas was soon observed, and I was tempted to pursue the disease wherever any recent erythema showed itself, with this disastrous result.

On these cases an advancing edge of more resistant lupus is seen as a rule. In this edge most of the recurrences will be seen in cases which have done well, and it is necessary to treat again and again until no fresh developments arise. On this account it is impossible to call a case cured until years have elapsed.

The nodular forms without much erythema do well with the light as a rule. In some cases, however, the good results are slower to arrive than in others, and I have observed that the factor of general health is a large one. It would seem that the reaction to the disease in these cases is not sufficient to produce the desirable erythema, which is undoubtedly reparative. Even when this is artificially supplied the leucocytic invasion is not sufficient. One case of this order was of a very delicate strumous type, while a second had phthisis. In the first relief of the disease was produced with some tendency to recur in spots, in the second very little could be done. One spot only seems to have disappeared, while others were removed surgically, being in places where the scars would not show. In cases where the general health is satisfactory what erythema is present at first dies away on the subsidence of that caused by the treatment, and the nodules pale till they are no more than the colour of freckles, and many disappear from view altogether. But it is not infrequent that nodules are left behind, inveterate and persistent, and which only the longest course of treatment might be expected to relieve. On the other hand, the nodules are clearly demonstrated as the focus of the disease, and the simple and not very painful process of stabbing them with a pointed match-stick dipped in pure carbolic acid will get rid of them, even if it leaves behind small pitted scars. This is the course I adopt now when I have to deal with any of these inveterate and chronic cases. It may certainly be claimed for radio-therapy that it decreases the amount of the area to be treated with caustics eventually, while in many cases of these inveterate forms it relieves without resort to caustics at all.

In the hypertrophic forms it is seen at once what an enormous advantage radio-therapy can offer. With the large masses of granulation tissue exuberant, foul and greatly disfiguring, the temptation to remove the whole with the sharp spoon is almost irresistible. If this is performed great loss of tissue with irregular deep scarring will occur, whereas the X-rays soon have a marked effect upon the granulations, levelling them down to the skin, and leaving a scar in no way particularly noticeable except for occasional development of telangiectatic vessels, which have in a few cases made a permanently red colouration of the scar. This is possible after any prolonged X-ray treatment, and not dependent on the kind of disease treated.

After the X-rays have reduced the hyertrophied granulation masses, there may appear ordinary lupus nodules which persist. These are not as a rule difficult to deal with, either by continuing the rays or with the light lamp.

The good effect of a course of the rays will continue for a long while after the rays are stopped. It is my practice to go very slowly in these cases, to prevent if possible the development of telangiectases by too rapid treatment. The result, as proved by time, seems to be very satisfactory as far as my experience goes ; but if ever any part begins to develop, for instance at the edge of a patch or in a fresh part of the body, the production of granulation material is extraordinary in its rapidity and amount.

In the ulcerated forms we have again an opportunity for the X-rays to show an advance in therapeutic methods. Large, deep and very chronic ulcers yield to their influence without added loss of tissue or the contractures which might be expected. Not only do they heal, but they remain healed. In very extensive cases of the disease, where little else could be accomplished, the complete healing of the sores has led to comfort and material well-being of the patients. This good result can be obtained on the mucous membranes as well as on the skin, provided the parts can be reached by the rays without an undue exposure of nearer parts. It is not advisable to treat the recesses of the nose with the rays from an X-ray tube, as the tip and alæ of the nose will show evidences of an overdose before good results can be obtained internally. The common site of perforation and ulceration within reach of the finger can be so treated and effectually. For internal use X-rays can be applied by means of high frequency vacuum tubes, which emit X-rays. I have tried this method with only partial success.

In verrucous forms there is a hard, warty growth of the corneous layer over each of the lupus nodules. The rays cause the corneous layer to shed, and the nodules exposed thereby are more easily dealt with, by other methods if desired, or by the continuation of radio-therapy. Some very good results have occurred from X-rays alone.

In œdematous forms the X-rays cause the œdema to subside, and in this particular I think they supersede all other forms of treatment. Occasionally the lymphstasis is out of all proportion to the apparent amount of lupus present. The lips, if involved, hang pendulous and unprotected ; they ulcerate and become useless. The leg, if involved, looks as if the subject of elephantiasis ; but the œdema subsides and lupus nodules, which previously were not obvious, become visible and can be treated.

In sclerodermic, hard and indurated forms, the X-rays soften the induration, heal any incidental ulcers, and, as far as my experience goes, the results are lasting.

*Conclusions.*—For the most part I have obtained better results with the X-rays than with light ; but I believe that the Finsen lamp is the better radio-therapeutic agent for nodular lupus, while the X-rays are better for hypertrophic, ulcerated, verrucous and sclerodermic forms.

Recurrences occur in this as in other treatments, and the cases must be jealously watched at intervals after they are discharged relieved. But it can be claimed that there is not the loss of tissue attendant upon the other treatments, and consequently not a tithe of the disfigurement resulting. On the parts of the body beneath the clothing, where possible, excision is probably the best available as well as the most rapid method. On the parts that show, radio-therapeutic measures, supplemented by carbolic acid puncture if required, will give slow but the best results.

ANALYSIS OF EIGHTY CASES OF LUPUS VULGARIS.

| | | | |
|---|---|---|---|
| 1. | Patients in whom spots have been relieved, with no recurrence in situ, though lupus may be present in mucous membranes, or on other parts of the body .. .. .. .. .. .. .. | 20 = | 25% |
| 2. | Patients discharged with great improvement .. | 15 = | 18.75% |
| 3. | Discharged with some improvement (ulcerations healed, &c.) .. .. .. .. .. .. | 9 = | 11.25% |
| 4. | Improved ; still under treatment .. .. .. | 11 = | 13.75% |
| 5. | Ceased treatment prematurely .. .. .. | 8 = | 10% |
| 6. | No improvement .. .. .. .. .. .. | 6 = | 7.5% |
| 7. | Recurrence after apparent relief .. .. .. | 3 = | 3.75% |
| 8. | Fresh development in vicinity of healed patch .. .. | 2 = | 2.5% |
| 9. | Dead .. .. .. .. .. .. .. | 6 = | 7.5% |

| NAME. | AGE. | FIRST TREATMENT. | LAST TREATMENT. | NATURE OF TREATMENT. | POSITION. | RESULT. | NOTES. | CLASS. |
|---|---|---|---|---|---|---|---|---|
| E. H. | 21 | Dec.,'01 | X-rays, Mar.'03 ac. lac. Apl.,'03 | Light, examined by X-rays later. Ac. lactic gums and m.m. .. .. .. | Nose, upper lip, cheeks, nose ulceration, contraction, ulceration of upper lip, gums, palate | Relieved .. .. .. .. | Nodular type, no previous suggested treatment. New nose by Mr. Groves | I. |
| W. R. | 17 | Dec.,'01 | Jan.,'04 | Light and X-rays | Nose, alæ and tip; right hand, back, left arm by elbow, left foot | Relieved mainly .. .. | Erythematous type mainly. Now returned with a patch on elbow. Had a burn on foot | VIII. |
| F. B. | 20 | Dec.,'01 | Feb.,'02 | Light only .. .. | Left cheek, patch size of a sixpence | Relieved .. .. .. .. | Nodular; one year duration only | I. |
| W. H. | 15 | Dec.,'01 | July,'04 | Light, few X-rays | Face, entire; ears, neck, extending to chest and shoulders | No improvement .. .. | Ten years duration. Erythematous type; couldn't tolerate X-rays | II. |
| V.H. | 19 | Dec.,'01 | Feb.,'02 | Light only .. .. | Tip of nose, m.m. in nose | Well, except ulceration internally | Nodular .. .. .. | I. |
| M.H. | 40 | Dec.,'01 | 1906 | Light, X-rays, ac. carb. | Nose, face generally | Great improvement with disfigurement | Nodular; a very long-standing, deep infection | II. |
| E.H. | 27 | Dec.,'01 | Jan.,'06 | Light .. .. .. | Face, nose, lip .. .. | Improved, but no permanence in some patches | Phthisis, nodular; pitting and scarring | III. |
| E.H. | 52 | Dec.,'01 | Mar.,'05 | Light .. .. .. | General on face .. | Dead of carcinoma mammæ subsequent to operation | | IX. |
| E. M. | 7 | Jan. '02 | Nov.,'06 | Light mostly, some X-rays | Right cheek, left thigh | Healed face and thigh, recurrent | Nodular since infancy; recurrence on edge of scar of thigh | VII. |
| J. D. | 53 | Jan.,'02 | Oct.,'02 | Light .. .. .. | Bridge of nose .. .. | Healed, but not sound in 1903 | Erythematous | III. |

| NAME. | AGE | FIRST TREATMENT. | LAST TREATMENT. | NATURE OF TREATMENT. | POSITION. | RESULT. | NOTES. | CLASS. |
|---|---|---|---|---|---|---|---|---|
| J. W. | 32 | Jan.,'02 | Oct.,'06 | Light and X-rays | Whole face ; neck to chest | Dead of carcinoma, angle of mouth, after operation by Mr. Groves for removal, and plastic to lip | Erythematous variety, very long duration and extensive. Improved under treatment considerably | IX. |
| E. B. | 56 | Jan.,'02 | July,'06 | Light and X-rays | Face, entirely ; ears | Improved, but fails to check development | Nodular and pigmented ; very deep. Nose plastic by Mr. Greig Smith, affected now | III. |
| A. L. | 37 | Jan.,'02 | Apl.,'04 | Light, X-rays, radium | Right cheek, extending over nose | No improvement .. .. | Erythematous ; absolutely nothing was done for this case by any form of treatment. 25 years duration | VI. |
| M. B. | 19 | Jan.,'02 | Jan.,'04 | Light, X-rays .. | Tip, and right side of nose, right cheek, left upper lip | Relieved .. .. .. .. | Hypertrophic .. .. | I. |
| M. G. | 19 | Jan.,'02 | Nov.,'02 | Light .. .. .. | Beneath chin, and jaw both sides | By letter from S. Africa, "keeping well" | Nodular .. .. .. | II. |
| M. B. | 31 | Jan.,'02 | Recent | Light .. .. .. | Left cheek, right chin, eyelids | Recrudescent, occurs in spots | Nodular ; many operations previously | IV. |
| B. B. | 36 | Jan.,'02 | May 7th '02, returned during last few days | Light no good ; 18 X-rays | Huge ulceration of alæ, septum and upper lip | Well within reach of rays, but m.m. beyond uncertain | Left prematurely, but good effects went on for a long while afterwards | IV. |

| | | | | | | | | |
|---|---|---|---|---|---|---|---|---|
| J.E.B. | 70 | Feb.,'02 | Jan.,'03 | Light .. .. .. | Left cheek and nose | Improvement .. .. .. | 7 years' treatment. Erythematous ; left prematurely owing to illness at home | V. |
| P. R. | 20 | Apl.,'02 | May 30, 1903 | Light, X-rays, ac. lactic m.m. | Right cheek, upper lip and gums | Well .. .. .. .. .. | Dactylitis operation | I. |
| J. L. | 17 | Mar.,'02 | Jan.,'03 | Light, no imprvmt. | Whole face .. .. | .. | Hypertrophic .. .. | IV. |
| | | Feb.,'05 | Present | X-rays .. .. .. | Neck .. .. .. .. | Great improvement .. | Great destitution at first ; Jan. '03, no improvement ; then workhouse, Feb., '05 | |
| W. P. | 9 | Apl.,'02 | May,'03 | Light .. .. .. | Face .. .. .. .. | Some improvement .. | Great improvement. Left prematurely ; nodular type | V. |
| H. S. | 4½ | Apl.,'02 | Aug.,'04 | Light only .. .. | Right cheek .. .. | Two small recurrences .. | Nodular .. .. .. | VII. |
| | | Apl.,'06 | July 7th 1906 | | | | | |
| E. L. | 40 | Apl.,'02 | Mar.,'05 | Light, radium, X-rays | Whole face .. .. | Practically well .. .. | Great scarring through operation ; ulceration, destruction of nose, great disfigurement | II. |
| R. A. | 46 | Apl.,'02 | Sep.,'03 | Light and X-rays | Nose, m.m. ; whole face | Improvement .. .. .. | Ulcerations 12–14 yrs., operation 11–12 times, attended irregularly | VI. |
| G. F. | 14 | Mar.,'02 | June,'04 | Light, X-rays, ac. lactic | Tip of nose, extending from m.m., involved heavily | Well externally, but internally not | Nodular, externally ; m.m. involved to back of pharynx | I. |
| M. W. | 23 | Feb.,'03 | Jan.,'05 | Light, ac. lactic, int. | Tip of nose, m.m. nose | Cured externally, not internally | .. | I. |
| S. N. | 59 | Mar.,'03 | Sep.,'03 | Light only .. .. | Both cheeks and nose 13–14 patches | As bad as ever, Nov., '06 | Patches scraped .. .. | VI. |
| O. L. | 17 | Mar.,'03 | Apl.,'04 | Light. Permang. of pot. painting ; to increase Rn. X-rays at end | Thigh, neck, ear .. | Some improvement when last seen | No answer to card | III. |

| NAME. | AGE | FIRST TREATMENT. | LAST TREATMENT. | NATURE OF TREATMENT. | POSITION. | RESULT. | NOTES. | CLASS. |
|---|---|---|---|---|---|---|---|---|
| C. P. | 65 | Apl.,'03 | May,'04 4 X-rays, May,'05 | Light, X-rays at end | Left side face, under chin | Improvement | Scarring variety | III. |
| E. W. | 36 | Apl.,'03 | Aug.,'03 | Light only (Pyrogallic ac. carbol. to increase Rn.) | Left forefinger | Well | Well Nov., 1906 | I. |
| E. B. | 57 | May,'03 | Mar.,'05 | X-rays mainly | Both cheeks, under chin, extensive | Great improvement, most of it cured | Slight burn, right cheek, with slight teliangiectasis | III. |
| F. Y. | 26 | May,'03 | June,'04 | .. .. .. .. | Centre of right cheek | Improvement, ? well; writes sometimes, place hardly noticeable; gets red at times, except at upper part | Nodular L. | III. |
| A. F. | 21 | May,'03 | Rec'ntly to ankle (ac. carbol.) | Light and X-rays ac. carbol. and X-rays ankle | Extensive on cheeks right wrist, ankle | Face probably well, wrist a little doubtful in one part. Ankle too recent | Nodular, L. | I. |
| E. B. | 21 | June,'03 | Rec'ntly | Light to Jan., '03, X-rays after, ac. carb. to recurrent nodules | Cheek and nose | Excellent, though one or two spots are uncertain | Lupus Hypertrophicus, very bad | III. |
| M. D. | 34 | Aug.,'03 | Ceased attendance after three weeks | .. | .. | .. | .. | V. |
| C. F. | 31 | Aug.,'03 | Aug.,'05 | X-rays | Thumb | Healed, Nov., 1906 | Verrucous | I. |
| C. H. | 23 | Oct.,'02 | Rec'ntly | Light, X-rays, ac. carb. to promote H.F. internally | Extensive on face, cheeks, lip, nose, eyelids; m.m. nose | Healed mainly; some doubtful points persist in eyelids and on cheek | Was scraped 11 times before light treatment; nodular type | IV. |
| F. L. | 28 | Oct.,'02 | Mar.,'06 | Light only | Centre right cheek, size of half a crown | Well at present | Nodular type. Recurred Jan., '06, after apparent relief since May, 1904 | I. |

| | | | | | | | | |
|---|---|---|---|---|---|---|---|---|
| M. B. | 42 | Oct.,'02 | Jan.,'06 | Light only .. .. | Extensive left cheek | Well, except for fresh development in left eyebrow | Nodular; scraped 23 years ago, 3 times | VIII. |
| H. M. | 10 | .. | .. | .. | .. | .. | Lost sight of .. .. | IX. |
| E. G. | 27 | Dec.,'02 | Feb.,'04 | Light at first, then X-rays only | Left leg and thigh, foot; could scarcely walk at all | Œdema has improved so much that he can walk freely. A few scattered L. nodules present, for which he has returned for treatment | Œdematous type, very slight appearance of nodules | IV. |
| C. B. | 26 | Dec.,'02 | Mar.,'c6 | X-rays mainly .. | Cheeks, nose, chin, neck to clavicles | Great improvement | A very severe nodular case, with a few ulcerations | II. |
| A. L. | 47 | Jan.,'03 | Nov.,'03 | Light only .. .. | Right cheek, extensive under chin, right forearm | Practically nil. Improvement at time apparently considerable | Very extensive in various parts of body. On returning home fresh developments in parts previously untouched | VI. |
| C. T. | 67 | Mar.,'04 | July,'04 | X-rays mainly .. | Remains of nasal cartilages and septum, face extensive, cheeks and forehead | Ulcerations healed .. .. | Very ulcerated; nose gone | II. |
| M.A.S. | 60 | Mar.,'04 | Aug.,'05 | X-rays .. .. .. | Huge ulcer right cheek, nose ulcerated in three places; right ear, cheek and nose | Ulceration healed, and remaining so | Nov., '06, " no further spread." Ulceration very extensive, ulcers several square inches in extent. Hip disease at 14, lame now. Blind right eye. Erythematous variety | II. |

| NAME. | AGE | FIRST TREATMENT. | LAST TREATMENT. | NATURE OF TREATMENT. | POSITION. | RESULT. | NOTES. | CLASS. |
|---|---|---|---|---|---|---|---|---|
| M. H. | 34 | Apl.,'04 | Oct.,'04 | X-rays . . .. | Nose, tip ; left foot, dorsum and sole of 3rd toe, and metatarsal and on shin | Nose well, foot recurrent | Verrucose, nodular, ulcerated. 2nd toe removed by Mr. Groves before '04 and scraping ; 3rd toe removed by Mr. Groves before July, '06 ; duration 20 years on face. One spot healed under Dr. Harrison's double lotion treatment. Recurrence in foot | I., IV. |
| R. J. | 57 | Apl.,'04 | Nov.,'05 | .. .. .. .. | Very extensive on face | Great improvement, and this still continues (Dr. Harrison) | Nodular type .. .. | II. |
| W. B. | 34 | June,'04 | June 6, 1906 | Light and X-rays | Ulceration of septum, nose and alæ, and upper lip ; thickening of upper lip | Well .. .. .. .. .. | Nov. 18th, '04, seemed well ; slight indefinite recurrence, April 7th,'06, which yielded readily | I. |
| C. J. | 65 | June,'04 | Oct.,'05 | Light and X-rays | Over whole face ; ulcerated .. .. .. | Ulcerations well, and keeping so ; nodules do not disappear | Nodular type. Has recently returned for treatment. Nodule no longer confluent ; very discrete now. | IV. |
| J. D. | 57 | June,'04 | Sep.,'06 | X-rays only ; coc. and lactic ac. internally | Edges of nostrils ulcerated, m.m. involved | Healed externally, doubtful internally | Ulcerated type. Lives in country; occasional return | II. |

| | | | | | | | | |
|---|---|---|---|---|---|---|---|---|
| W. C. | 21 | July,'02 | Mar.,'05 | Light and X-rays | Face, and below chin on neck | Improvement to skin | Nodular type. Tubercular spinal (cervical) caries | II. |
| L. G. | 1 | Jan.,'04 | June,'06 | Light at first, X-rays later | Shin and elbow .. | Improvement .. .. .. | Nodular .. .. .. | II. |
| B. H. | 74 | Apl.,'04 | Nov.,'05 1 or 2, '06 | X-rays .. .. .. | Face .. .. .. .. | Two ulcers healed entirely, at angle of mouth not quite healed | Chronic ulceration for years | II. |
| F. W. | 3 | Sep.,'04 | July,'05 | Light only .. .. | Small patch, right cheek | Healed .. .. .. .. | Nodular type .. .. | I. |
| E. C. | 20 | Sep.,'04 | July,'06 | Light, X-rays later | Under ears and chin | Some improvement possibly | Very scaly, nodular and erythematous ; very resistant to treatment | VI. |
| A. S. | 25 | July,'04 | Sep.,'06 | Light no good, X-rays | Neck .. .. .. .. | Improvement with X-rays | L. Nodules round scar on neck. Light useless, owing to soft parts ; no pressure possible | IV. |
| M. M. | 60 | Nov.,'04 | May,'05 | X-rays only .. .. | Cheeks and nose, extensive | Improvement .. .. .. | Nodular type | III. |
| W. W. | 20 | Feb.,'05 | Mar.,'06 | Light and X-rays | Face, eyelids, cheeks ; extensive deficiency in septum | Much improved. Few spots not well | Very scarred ; has had X-rays in London with burns, with improvement '01 and '02. Scraped 8 times , 5 years since last scraping. Radium was tried without effect | II. |
| E. W. | 30 | Jan.,'05 | Aug.,'06 | X-rays, ac. carbol. | Face, nose, lip, right temple, under chin, left cheek | Great improvement, one or two spots left only | Increased T.V.F. right apex, no *râles* | IV. |

| NAME. | AGE | FIRST TREATMENT. | LAST TREATMENT. | NATURE OF TREATMENT. | POSITION. | RESULT. | NOTES. | CLASS. |
|---|---|---|---|---|---|---|---|---|
| E. B. | 9 | Feb.,'05 | .. | Cautery and ac. carbol. only ; no radio-therapy | Chest and neck .. | Chest well, neck no improvement | Nodular patches small | IV. |
| M. B. | 22 | Mar.,'05 | Present | X-rays only. Ac. carbol. increased reactions | Backs of both hands, and feet, inner side arm, under chin | Very great improvement, but fresh place on arm developed since rest period | Hypertrophic .. .. | IV. |
| P. H. | 5 | Apl.,'05 | June,'06 | X-rays, ac. carbol. | Cheek and jaw .. | Healed ; hardish still on jaw | Lupoid .. .. .. | I. |
| H. P. | 23 | May,'02 Nov.,'06 | Sep.,'03 | Light, X-rays lately | Margin of nose, m.m., left cheek | Nose healed, cheek not | Ulcerated, nodular ; left, but failed to report ; has returned with cheek diseased again | V. |
| M. B. | 36 | May,'02 | Sep.,'04 | Light, X-rays and ac. lactic | Tip of nose, m.m. .. | Externally well, m.m. not | Nodular, m.m. scraped lately | I. |
| J.McC. | 18 | May,'02 | .. | .. | Ulceration ; mouth, eyes | Nearly well .. .. .. | Died of Empyema ; very undersized and poor ; workhouse child | IX. |
| J. M. | 54 | July,'02 | May,'05 | Light and X-rays | Tip of nose and alæ | Well .. .. .. .. | Very ill with cholelithiasis, nodular type | I. |
| S. A. | 58 | July,'02 | Oct.,'03 | Light and X-rays | Face and nose ulcerated | Great improvement .. | Dead of renal disease. " No trace of disease on face at death." | IX. |
| H. P. | .. | .. | .. | .. | .. | .. | Discharged for irregularity of attendance | V. |
| F. P. | 18 | July,'02 | July,'04 | Light, very few X-rays | Face and nostrils .. | Seemed well on face .. | Operated previously ; dead of acute nephritis | IX. |

| | | | | | | | | |
|---|---|---|---|---|---|---|---|---|
| F. H. | 30 | Aug.,'02 | Oct.,'02 | Light only .. .. | Face, entire; lips enormous and ulcerated | Improvement, some .. | Too short a stay for lasting improvement | V. |
| M. B. | 15 | Aug.,'02 | Dec.,'05 | Light and X-rays | Face, buttock, arm .. | Well on face and buttock; one small recent suspicious spot on arm | Hypertrophic; worst case. L. Sclerodermica on buttock; dactylitis, both hands | I. |
| A. L. | 60 | Aug.,'02 | Dec.,'02 | Light .. .. .. .. | Face .. .. .. .. .. | No relief possible | Left too soon for any result | V. |
| B. H. | 13 | Sep.,'02 | Mar.,'05 Recent examination, '06 | X-rays, ac. carb. ext. nose | Nose .. .. .. .. .. | Ulcerated nose cured, recent lupus on side of nose | .. | I. |
| F.R. | 32 | Apl.,'05 | July,'05 | Light, ac. lactic ext. .. | Lip, m.m., nose .. | Healed externally .. .. | M.m. improved .. .. | I. |
| J. H. | 29 | May,'05 | July,'06 | X-rays, light later | Neck .. .. .. .. .. | Improved and cured .. | .. | II. |
| A, R. | 10 | May,'05 Jan.,'06 | Aug.,'05 Sep.,'06 | X-rays .. .. .. .. | Nose, lip .. .. .. .. | Great improvement .. | Nodular .. .. .. .. | VII. |
| E. W. | 59 | May,'05 | Nov.,'05 | X-rays .. .. .. .. | Face, nose, m.m. .. | Recurrence .. .. .. | Nodular, face, m.m. improved | I. |
| C. D. | 22 | Aug.,'05 | May,'06 | Light and X-rays | Front of right ear and under chin | Apparently well; m.m. doubtful | Phthisis and anæmia; nodular | II. |
| W. V. | 30 | Sep.,'05 | May,'06 | Light only .. .. | Left cheek .. .. | ? well, looks so.. .. .. | Left too early, and any improvement lost. Vertigo | V. |
| L. J. | 29 | Oct.,'05 | Apl.,'06 | Light mainly, X-rays | Left cheek .. .. | No disease visible, Nov., 1906 | X-rays. Previously to B.G.H. great irritation; nodular | II. |

